# Kinematics-Based Predictions of External Loads during Handcycling

**DOI:** 10.3390/s24165297

**Published:** 2024-08-15

**Authors:** Griffin C. Sipes, Matthew Lee, Kellie M. Halloran, Ian Rice, Mariana E. Kersh

**Affiliations:** 1 Department of Mechanical Science and Engineering, University of Illinois Urbana-Champaign, Urbana, IL 61801, USA; gsipes2@illinois.edu (G.C.S.); ml95@illinois.edu (M.L.); kellie2@illinois.edu (K.M.H.); 2 Department of Health and Kinesiology, University of Illinois Urbana-Champaign, Urbana, IL 61801, USA; ianrice@illinois.edu; 3 Beckman Institute for Advanced Science and Technology, University of Illinois Urbana-Champaign, Urbana, IL 61801, USA; 4 Carle Illinois College of Medicine, University of Illinois Urbana-Champaign, Urbana, IL 61801, USA

**Keywords:** handcycling, kinematic data, machine learning, neural networks, biomechanics, inertial measurement units, optical motion capture, spinal cord injury, external load prediction, temporal convolutional network (TCN)

## Abstract

The increased risk of cardiovascular disease in people with spinal cord injuries motivates work to identify exercise options that improve health outcomes without causing risk of musculoskeletal injury. Handcycling is an exercise mode that may be beneficial for wheelchair users, but further work is needed to establish appropriate guidelines and requires assessment of the external loads. The goal of this research was to predict the six-degree-of-freedom external loads during handcycling from data similar to those which can be measured from inertial measurement units (segment accelerations and velocities) using machine learning. Five neural network models and two ensemble models were compared against a statistical model. A temporal convolutional network (TCN) yielded the best predictions. Predictions of forces and moments in-plane with the crank were the most accurate (r = 0.95–0.97). The TCN model could predict external loads during activities of different intensities, making it viable for different exercise protocols. The ability to predict the loads associated with forward propulsion using wearable-type data enables the development of informed exercise guidelines.

## 1. Introduction

Full-time manual wheelchair users, such as persons with spinal cord injuries, are at increased risk of chronic diseases such as cardiovascular disease (CVD), diabetes, and metabolic syndrome [1,2,3]. Although the evidence supporting the benefits of physical activity is clear, many individuals with spinal cord injuries are physically inactive and sedentary leading to worsening symptoms and increased levels of disability [4,5]. Unfortunately, the transition to manual wheelchair use presents challenges as the arms become the source of all activities of daily living. For example, as many as 68% of manual wheelchair users have rotator cuff tendon tears [6]. An optimal exercise for improving cardiopulmonary function requires sufficient taxation of cardio-respiratory fitness levels without further increasing the risk of musculoskeletal injury. Current exercise recommendations for full-time manual wheelchair users have not resulted in significant changes in chronic disease, and this has been attributed in part to a lack of specificity in the prescription for manual wheelchair users who have less access to muscle mass during exercise. While activities that involve both pushing or pulling using the shoulder have been suggested [7], the specific exercise modes by which one should engage in such activities have not been definitively identified.

Handcycling has emerged as a promising exercise mode for wheelchair users [8] because the loads on the shoulder are lower than everyday wheelchair propulsion [9]. Exercise guidelines (intensity, frequency, etc.) for handcycling remain to be established and require assessment of both cardiovascular stimulation and musculoskeletal safety. Musculoskeletal assessments, such as shoulder torque, provide objective measures of shoulder function and require measuring the external hand loads. However, unlike the variety of commercially available means for measuring ground reaction forces during legged walking, there are no off-the-shelf options for direct measurement of hand loads during handcycling. Custom-built instrumented crank handles have been developed [9,10] but are not readily accessible outside of academic research settings. Enabled by the recent advances in wearable sensors that can measure accelerations and other kinematic variables, the field has turned towards the use of kinematic data to infer external loads via direct or indirect methods.

In the case of legged locomotion, the ground reaction force can be solved for directly using Newton’s 2nd law if the kinematics of major body segments are measured [11,12]. Both inertial measurement units and optical motion capture have been used to estimate ground reaction forces during walking, stair-use, and running [11,12,13,14,15]. However, tracking all segments can be difficult and may not be feasible for every activity, thus motivating the use of statistical or machine learning models to predict external loads from subsets of kinematic data [13,16].

Segment accelerations have been used in neural network machine learning models to predict the ground reaction forces during legged walking [13]. Glenohumeral joint reaction forces during wheelchair propulsion have been predicted using a combination of inertial measurement units and electromyography data in a bidirectional long short-term memory network architecture [16]. To our knowledge, no study has reported kinematic-based predictions of external loads during handcycling. Therefore, the objective of this study was to evaluate the capacity of machine learning models to predict hand loads using segment accelerations. The capacity to measure external loads using kinematic data available from wearable sensors would enable field-based measurements and negate the need for specialized equipment.

To mimic data that could be measured via an inertial measurement unit, we based our analyses on accelerations of the radius segment. We evaluated two classes of machine learning models (neural network and ensemble) and compared their prediction performance against a statistical regression model.

## 2. Materials and Methods

### 2.1. Dataset

**Figure 1:**
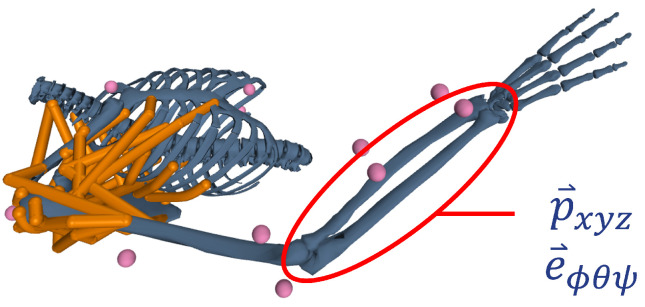
Motion-capture markers (pink spheres) used with subject-scaled rigid-body musculoskeletal model of upper arm. Radius segment (red circle) center of mass data derived from the radius position $\vec{p}$ and orientation $\vec{e}$ with respect to the global reference frame.

Biomechanics data of full-time wheelchair users (n = 20) completing a handcycling exercise protocol as part of a separate study [10] were used. For each participant, six propulsion cycles were extracted during moderate-intensity (45% peak power) handcycling and six cycles during high-intensity (90% peak power). A custom instrumented handle was used to measure the three forces and three moments applied at the right handcycle handle (sampling frequency = 2 kHz). Kinematic data of the right arm were recorded using a 10-camera motion capture system (Vicon, sampling frequency = 100 Hz). A rigid-body model [17] was scaled to each participant and used to determine the radius segment center of mass position and orientation using inverse kinematics (Figure 1). Data from each propulsion cycle were interpolated into 360 data points corresponding to the 360 degrees of crank rotation.

The segment positions were numerically differentiated twice using the central difference formula to obtain linear accelerations ($\vec{a}\left(t\right)$) (Equation (1)):

(1) $$\vec{a}\left(t\right)\;=\;\frac{\vec{p}\left(t+\tau \right)-2\vec{p}\left(t\right)+\vec{p}\left(t-\tau \right)}{\tau^{2}}$$

where $\vec{p}$ is the radius center of mass position, *t* is the current time, and τ is the interval between data points. Orientations (Euler angles) were differentiated once to obtain angular velocities ($\vec{\omega }\left(t\right)$) (Equation (2)):

(2) $$\vec{\omega }\left(t\right)\;=\;\frac{\vec{e}\left(t+\tau \right)-\vec{e}\left(t-\tau \right)}{2\tau }$$

where $\vec{e}$ is the vector of Euler angles denoting the radius center of mass in the global coordinate system.

### 2.2. Model Objectives and Inputs

The objective of all models was to predict the continuous 6-degree-of-freedom applied loads at the handcycle handle given kinematic and anthropometric data. The training data for all models were based on 90% of participants (n = 18, randomly selected) while data from the remaining two participants were used as the test set. Data for model validation were based on a further split of the training dataset using n = 2 participants’ data. Participant weight (lbs), wingspan (in), and exercise intensity were included with the kinematic data as model input features. Exercise intensity was defined as 0.5 or 0.9 to denote the varying protocol intensities. Prior to model training, each input feature was standardized by subtracting the mean and dividing by the standard deviation. This process was used to standardize the training, test, and validation datasets.

### 2.3. Model Overview and Descriptions

A classical statistical regression model and several machine learning models were compared, including five neural networks and two ensemble models (described below). Our choice of machine learning models was motivated by the work of others in predicting kinetic data [13,14,16] in addition to testing models of varying complexity in similar applications. A dense network was chosen because it is one of the simplest neural networks. Temporal convolutional convolutional networks (TCNs) have been used to predict times series data [18], and we chose to evaluate their performance along with a simpler convolutional neural network (CNN). Regression tasks have been performed with ensemble models such as a random forest (RF) algorithm [19] and the more complex RF-based gradient boosting machine (GBM).

Each of the neural network models was compiled using mean squared error as the loss function and the Adam algorithm as the optimizer (learning rate = 0.0001). Models were trained for a maximum of 1000 epochs with an early stop callback, which stopped training early if the loss function did not decrease by 0.01 in 50 epochs. A batch size of 16 propulsion cycles was used during training. Neural network and ensemble model hyperparameters were tuned using a heuristic approach beginning with default values prescribed by the machine learning libraries. Some model parameters, such as the number of layers in some models, were chosen to imitate models from the literature that were used for similar applications. Regularization was implemented using a heuristic approach to minimize model overfitting. All neural network models were created using Tensorflow [20], the random forest model was created with scikit-learn [21], and the gradient boosting model was created with XGBoost [22]. All models were implemented using Python (v 3.9).

Linear regression: elastic net linear regression model (scikit-learn [21]), herein referred to as “Linear”. The Linear model was trained with the least absolute shrinkage and selection operator (L1) and Tikhonov (ridge) (L2) regularization of coefficients. The Linear model parameters were α=0.003, ρ=0.5, and maximum training iterations = 2000. The model was fit by minimizing the objective function (Equation (3)):

(3) $$\min_{\vec{\beta }}\frac{1}{2n_{samples}}{∥X\vec{\beta }-\vec{y}∥}_{2}^{2}+\alpha \rho ∥\vec{\beta }∥+\frac{\alpha (1-\rho )}{2}{∥\vec{\beta }∥}^{2}$$

where $\vec{\beta }$ is the vector of coefficients, X is the design matrix of predictors, $\vec{y}$ is the vector of outputs, α is a constant that multiplies the penalty terms, and ρ is the ratio of L1 regularization to L2 regularization.

**Figure 2:**
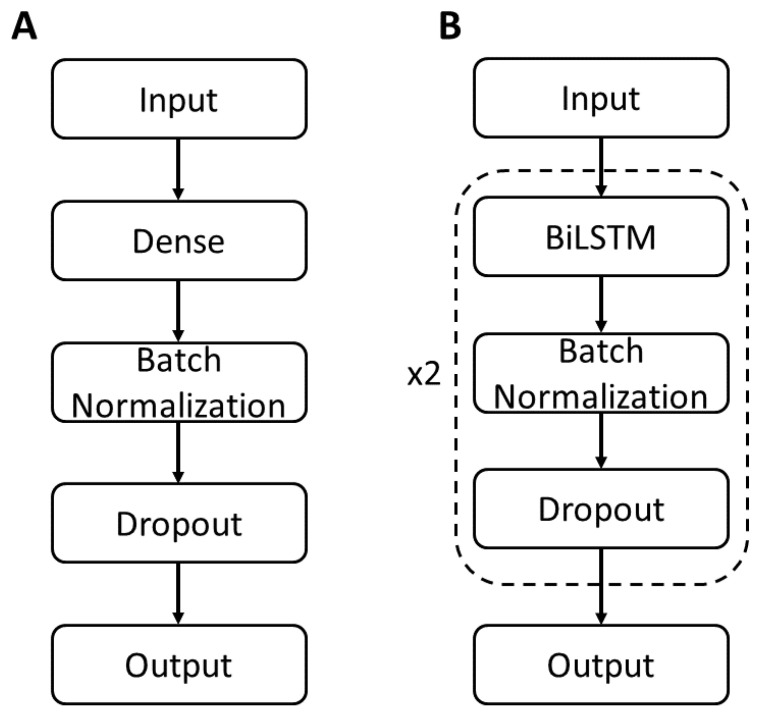
Neural network model architectures: (**A**) Dense. (**B**) Bidirectional long short-term memory (BiLSTM). Model parameters shown in Table 1.

Dense: dense neural network (Figure 2A) model with one hidden dense layer containing 200 nodes and L1/L2 regularization of 0.00001 applied to the kernel weights matrix. Batch normalization and dropout (45%) were applied after the hidden layer. The output layer was a dense layer with six nodes denoting the six output features. Model parameters for each of the neural network models are shown in Table 1.

Bidirectional long short-term memory (BiLSTM): two-layer BiLSTM model (Figure 2B). Each BiLSTM layer had 200 nodes with L1/L2 regularization (0.001) on the kernel weights matrix, recurrent kernel weights matrix, and bias vector. After each BiLSTM layer, batch normalization and dropout (45%) were applied. The output layer was a dense layer with six nodes.

**Figure 3:**
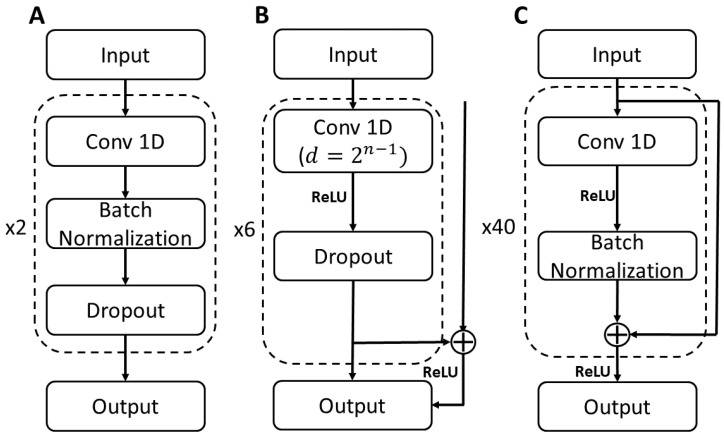
Convolution neural network architectures: (**A**) Convolutional neural network (CNN). (**B**) Temporal convolutional network (TCN). (**C**) Residual neural network (ResNet). Model parameters shown in Table 1.

Convolutional neural network (CNN): two 1D convolutional layers (Figure 3A) were used to mimic the structure of the BiLSTM model. Each convolutional layer had 200 filters, a kernel size of 64, causal padding, rectified linear unit (ReLU) activation function, and L1/L2 kernel regularization (0.00001). After each convolutional layer, batch normalization and dropout (50%) were applied. The output layer was a time-distributed dense layer with six nodes.

Temporal convolutional network (TCN): six hidden layers with $2^{n-1}$ dilation rate, where *n* is the layer number (Figure 3B). Each layer had 64 filters with a kernel size of 32 and L1/L2 regularization (0.01). After each layer was a ReLU activation layer followed by a dropout layer (40%). The output layer was a time-distributed dense layer with 6 nodes and L1/L2 kernel regularization (0.01).

Residual neural network (ResNet): forty layers of residual blocks (Figure 3C). Each residual block was made of a 1D convolutional layer starting with 32 filters and a kernel size of 32. The kernel size in the convolutional layer doubled every five layers. The convolutional layers used ‘same’ padding and had L2 regularization (0.001). After each convolutional layer, batch normalization and a ReLU activation layer were used. The output layer was a time-distributed dense layer with 6 nodes and L2 kernel regularization (0.001).

Random forest (RF) and gradient boosting model (GBM): two ensemble models were evaluated (Table 1). A random forest regressor [21] model was created with 400 decision trees, with each tree having a maximum depth of 10 levels. The second ensemble model was a gradient boosting machine (GBM) implemented using XGBoost [22]. This model sequentially built 2000 decision trees, each with a maximum depth of 10, using a learning rate of 0.01. It employed both row and column subsampling at 70% and included L1 and L2 regularization (both set to 0.2). The GBM was trained for 1000 boosting rounds.

### 2.4. Statistical Analysis

For each of the 24 propulsion cycles in the test set, the median absolute percentage error (MdAPE) was calculated for each kinetic degree of freedom predicted. This analysis was repeated for the eight models tested. The Linear model was used as the baseline for comparison as it was the simplest model tested.

The distribution of model errors was non-normal for some degrees of freedom based on Shapiro–Wilkes tests for normality. Therefore, for each degree of freedom, a Kruskal–Wallis test was used to determine whether the errors differed between the models tested. If the differences were significant, Dunn’s test for multiple comparisons was used to determine which models differed in performance compared with the Linear model.

The correspondence of the model prediction to the ground truth was also compared qualitatively by evaluating the shape of the predictions and quantitatively using Pearson’s correlation coefficients. Kruskal–Wallis tests were used to determine whether the distributions of correlation coefficients for each degree of freedom differed between models, and a post hoc Dunn’s test was used to determine differences compared to the Linear model.

Wilcoxon rank sum tests were used to compare predictions made by the best-performing model between the moderate and high-intensity propulsion cycles for each of the output features. Statistical analyses were performed using the SciPy package [23] in Python.

## 3. Results

Overall, errors in predictions of kinetics in the x- and y-directions, which contribute to the tangential and radial loads during handcycling, were lower (range: 32 to 56%) than kinetics in the out-of-plane z-direction (range: 45 to 101%). The Dense and RF models had significantly worse prediction performance (errors of 101 and 100%, respectively) than the Linear model for $F_{z}$ (66% error), and the RF model predictions were also significantly worse for $T_{z}$. Most of the neural network models performed significantly better (error range: 38–42%) than the Linear model (error = 54%) for predicting $F_{x}$ (Table 2). The ResNet and TCN models performed better than the Linear model for $F_{y}$ (errors of 38 and 36%, respectively) and $T_{x}$ (errors of 32%). The GBM model predicted better than the Linear model for $T_{y}$ only (error = 39%). There were no other significant differences in prediction error compared to the Linear model.

**Figure 4:**
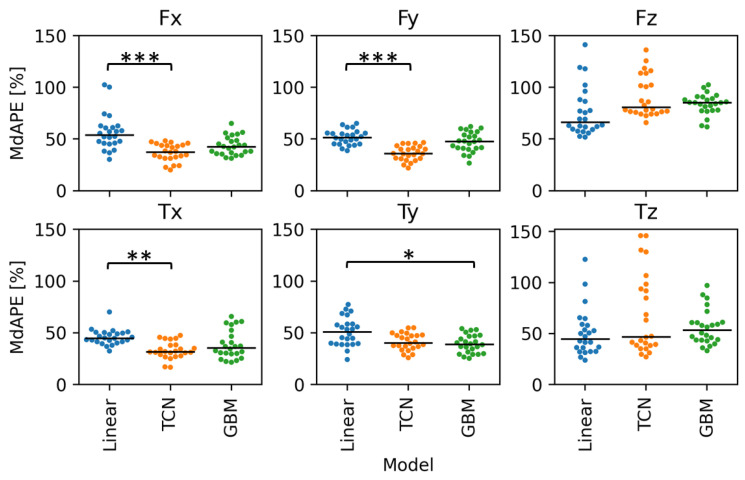
Comparison of model prediction (Linear, temporal convolutional network (TCN), and gradient boosting machine (GBM)) median absolute percentage error (MdAPE) for each output feature across the 24 propulsion cycles of the test set. *** p<0.001, ** p<0.01, * p<0.05.

Of the neural network models, the TCN model had the lowest overall error, and the GBM model had the lowest error of the ensemble models. The range of error values for the best statistical, neural network (TCN), and ensemble (GBM)) models was smaller in the x- and y-directions compared with the z-direction (Figure 4).

**Figure 5:**
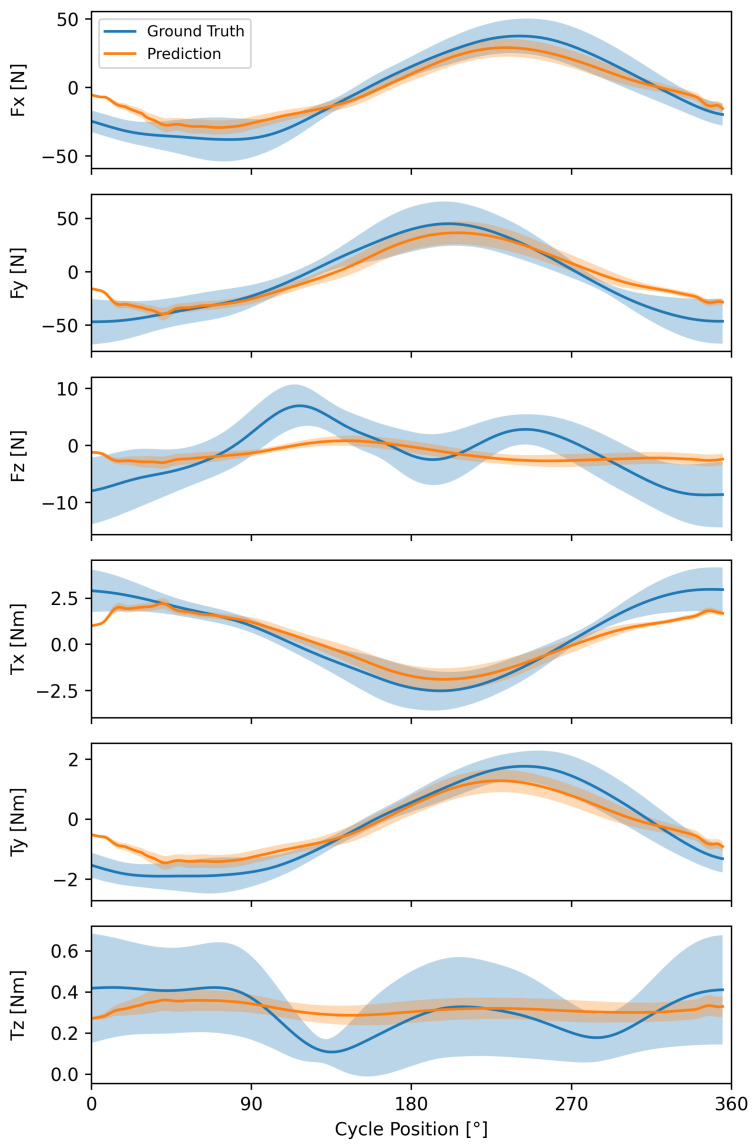
Average ground truth and average prediction of a propulsion cycle from the temporal convolutional network (TCN) model across each output feature. Predictions are colored orange and ground truth values are colored blue. Standard deviation ribbons are shown.

**Figure A1:**
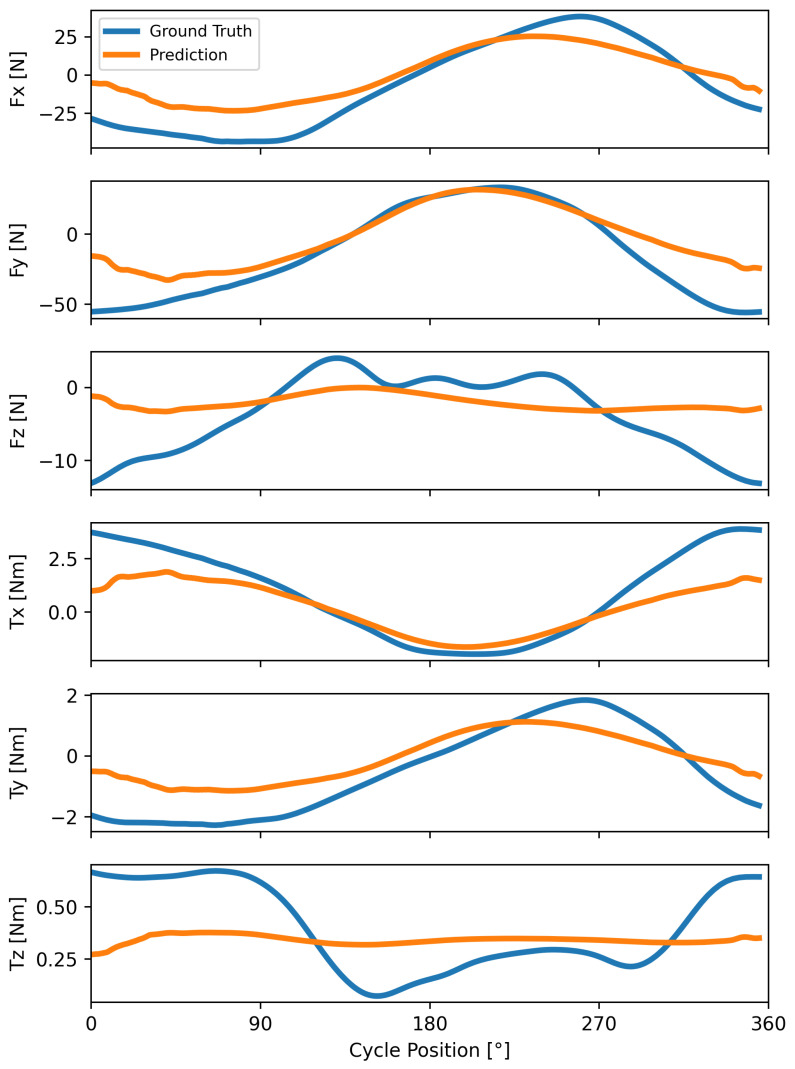
Representative (median error) propulsion cycle showing ground truth and temporal convolutional network (TCN) model prediction.

The shape of the average prediction from the TCN model matched well with the ground truth for the in-plane kinetic degrees of freedom (Figure 5 and Figure A1), with correlation coefficients ranging from 0.95 to 0.97 (Table 3). However, the model tended to underestimate the minima and maxima of each feature. For the x and y degrees of freedom, the errors tended to be highest at the beginning and end of the propulsion cycle. Force and torque profiles in the z-direction were poorly predicted.

**Figure 6:**
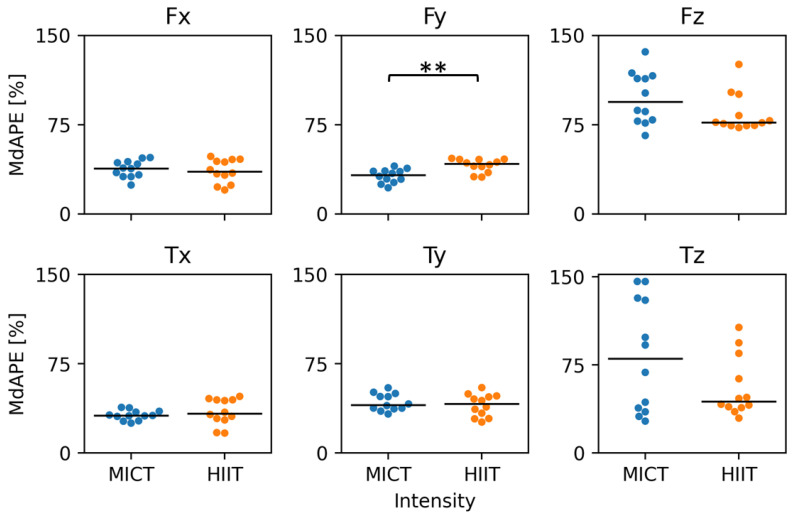
Comparison of temporal convolutional network (TCN) model prediction median absolute percentage error (MdAPE) between moderate-intensity (MICT) and high-intensity (HIIT) propulsion cycles for each output feature. **: *p* = 0.00323).

Finally, we evaluated whether model performance was sensitive to exercise intensity level. Errors in forces in the y-direction were moderately higher during high-intensity exercise (mean error = 41%) compared with moderate-intensity (mean error = 32%) (Figure 6). There were no significant differences in prediction performance between the two exercise intensities for the remaining degrees of freedom.

## 4. Discussion

We have shown that machine learning models can improve predictions of external loads during handcycling compared with a simpler elastic net linear regression model. Neural network models tended to perform better than ensemble models. Neural network predictions can leverage a longer time range of values, whereas tree-based ensemble models make instantaneous predictions at each timestep. Among the neural network models, convolutional neural networks outperformed recurrent (BiLSTM model) and dense neural network models.

Of all models tested, the predictive performance of the TCN was the most accurate for the kinetic parameters $F_{x}$ and $F_{y}$. During handcycling, most force is applied in the tangential and radial directions with respect to the crank center and is a combination of the $F_{x}$ and $F_{y}$ forces. In contrast, forces applied in the lateral direction ($F_{z}$) are lower in magnitude. Thus, while the lateral forces and torques had higher percent errors, the error magnitude was relatively small. The poor ability to predict lateral forces and torques may be due to the variability in propulsion technique (shaded blue regions in Figure 5) [10], despite the fact that handcycling is a closed-chain task and relatively constrained in terms of motion. Individual techniques have been classified for manual wheelchair propulsion but have not been used in handcycling. The inclusion of propulsion style, either as a categorical variable or via the inclusion of additional segment kinematics as inputs, in future machine learning models may prove useful for improving kinetic predictions. Another option for improving model performance is to tune the hyperparameters of the neural network via grid searching though requires significant computational resources. Finally, although there were some differences in prediction performance between moderate-intensity and high-intensity propulsion cycles, a single model is likely sufficient for multiple exercise intensities provided the intensity data are used as an input to the model.

The prediction performance for handcycling loads was similar to other studies that predicted external loads from kinematic data during locomotion. Johnson et al. used a combination of optical motion capture kinematics and inertial measurement unit data to predict the ground reaction forces and moments during various athletic maneuvers and reported Pearson’s correlation coefficients from r = 0.55 to 0.90 [13], similar to our results (r-range: 0.51 and 0.97) using the TCN model (Table 3). Liu et al. used joint angles from rigid body models to predict normalized ground reaction forces during stair use and reported Pearson’s correlation coefficients between r = 0.908 and r = 0.991 [14]. Normalizing the external loads reduces individual variability, which may increase model predictions, but is challenging to apply to data from persons with spinal cord injury.

There are a few limitations of this study. The size of the dataset used was relatively small, with 18 independent datasets used for training models. Remarkably, despite this small dataset, our best predictions were similar to others that used data from 80 subjects [14] for legged locomotion. One potential advantage of handcycling is the closed chain nature of the task which also may reduce the variability of the potential hand loads being predicted thus requiring less training data. The establishment of the minimum number of training datasets required for machine learning applications is the subject of ongoing research and may be task-dependent. Although the kinematic data used in this study were created to mimic that of a smartwatch-based inertial measurement unit, the data were derived from optical motion capture. Model hyperparameters were selected using a heuristic approach in this study to avoid the computational costs associated with alternative optimization methods such as the use of a grid search. While the use of a more advanced hyperparameter selection could be explored in future studies, we suggest that the inclusion of additional data or evaluation of alternative machine learning models should be prioritized to improve model accuracy.

## 5. Conclusions

The results of this study indicate that a TCN model could be used in place of an instrumented crank handle in future studies of handcycling propulsion biomechanics. However, it should be noted that the model is best suited for predicting loads that contribute to forward propulsion with more work needed to improve predictions in the lateral direction. Nonetheless, the ability to predict propulsive loads during handcycling now enables future research aimed at providing evidence-based exercise recommendations for people with spinal cord injuries by quantifying the external loads of different protocols. In addition to enabling musculoskeletal assessments of joint torques, the measurement of external loads is also needed to measure power during exercise. Power is among the most common metrics for assessing exercise effort. The machine learning model developed here now provides a means of assessing handcycling power using radius segment inertial measurements, such as those provided by a smartwatch. Measurements of power allow for research in the optimization of personalized exercise protocols (e.g., intensity, duration) based on an individual’s peak power output. Establishing relevant power levels is critical for the success of exercise interventions aimed at improving the health of people with spinal cord injuries. Importantly, the use of wearable-type data combined with machine learning predictions of hand loads allows for the assessment of handcycling outside of the lab environment.

Future research should validate the use of these models with inertial measurement unit data and investigate the feasibility of using such models to predict handrim forces during manual wheelchair propulsion. While inertial measurement units enable field-based data collection that can be recorded continuously during physical activity, more work is needed to predict long-term continuous kinetics without the need for parsing individual propulsion cycles as carried out in this study. With these efforts, field-based measurements of kinetics will enable the needed advances to improve the health of wheelchair users via exercise.

## Figures and Tables

**Table 1:** Machine learning model parameters. Models include: dense, bidirectional long short-term memory (BiLSTM), convolutional neural network (CNN), temporal convolutional network (TCN), residual neural network (ResNet), random forest (RF), and gradient boosting machine (GBM).

	**Neural Networks**		
	**Dense**	**BiLSTM**	
Layer type	dense	Bidirectional long short-term memory	
No. of layers	1	2	
Nodes per layer	200	200	
Regularization	L1/L2, 0.00001	L1/L2, 0.001	
Normalization	Batch normalization	Batch normalization	
Dropout rate (%)	45	45	
Output layer	Dense, 6 nodes	Dense, 6 nodes	
	**Convolutional Neural Networks**		
	**CNN**	**TCN**	**ResNet**
Layer type	1D convolutional	1D convolutional 2^(n − 1) dilation rate	1D convolutional
No. of layers	2	6	40
Filters per layer	200	64	32
Kernel size, padding	64	32	32, doubles every 5 layers
Padding	causal	N/A	same
Regularization	L1/L2, 0.00001	L1/L2, 0.01	L2, 0.001
Normalization	ReLU activation batch normalization	ReLU activation	ReLU activation batch normalization
Dropout rate (%)	50	40	N/A
Output layer	Time-distributed dense 6 nodes	Time-distributed dense 6 nodes	Time-distributed dense 6 nodes
	**Ensemble**		
	**RF**	**GBM**	
No. of estimators	400	2000	
Max. depth	10	10	
Booster	N/A	“gbtree”	
Objective	N/A	Regression, squared error	
Learning rate	N/A	0.01	
Subsampling	N/A	Row, column, 70%	
Regularization	N/A	L1/L2, 0.2	
No. of boosting rounds	N/A	1000	

**Table 2:** Comparison of model performance (median absolute percentage error (MdAPE)) across output features. Models include: linear, dense, bidirectional long short-term memory (BiLSTM), convolutional neural network (CNN), temporal convolutional network (TCN), residual neural network (ResNet), random forest (RF), and gradient boosting machine (GBM). Asterisks indicate models with errors significantly lower than the linear regression model.

Model Type	Model Name	Kinetic Prediction Error (MdAPE [%])					
		Fx	Fy	Fz	Tx	Ty	Tz
Statistical	Linear	53.97 (8.02)	51.65 (4.77)	66.36 (9.99)	44.84 (4.42)	51.05 (11.31)	44.86 (12.52)
Neural Network	Dense	41.72 (7.16) *	42.44 (24.87)	101.45 (22.24)	40.6 (10.31)	55.74 (9.2)	55.74 (20.42)
	BiLSTM	45.02 (6.22)	41.97 (12.07)	84.66 (14.89)	35.14 (10.55)	45.11 (7.28)	61.67 (19.5)
	CNN	39.83 (5.56) ***	43.18 (5.16)	85.64 (12.83)	40.27 (13.55)	45.32 (7.72)	51.53 (9.41)
	TCN	37.53 (6.26) ***	35.88 (5.17) ***	80.79 (6.66)	31.64 (4.31) **	40.44 (6.9)	46.79 (16.77)
	ResNet	41.58 (6.1) *	37.83 (7.58) **	88.82 (18.71)	31.96 (8.68) *	41.57 (6.88)	49.23 (8.85)
Ensemble	RF	47.35 (7.26)	43.35 (18.97)	99.53 (16.05)	41.65 (6.81)	49.45 (8.42)	65.4 (17.05)
	GBM	42.51 (6.36)	47.71 (6.73)	85.15 (5.72)	35.6 (8.12)	38.9 (7.92) *	53.45 (9.61)

*** p<0.001, ** p<0.01, * p<0.05. Data are expressed as median (median absolute deviation).

**Table 3:** Comparison of model performance (Pearson’s Correlation Coefficient (r)) across output features. Models include: linear, dense, bidirectional long short-term memory (BiLSTM), convolutional neural network (CNN), temporal convolutional network (TCN), residual neural network (ResNet), random forest (RF), and gradient boosting machine (GBM). Significance levels shown are compared with the linear regression model.

Model Type	Model Name	Output Feature Prediction Pearson’s Correlation Coefficient (r)					
		Fx	Fy	Fz	Tx	Ty	Tz
Statistical	Linear	0.81 (0.05)	0.86 (0.02)	0.78 (0.06)	0.89 (0.01)	0.84 (0.05)	0.3 (0.09)
Neural Network	Dense	0.92 (0.02) ***	0.95 (0.01) ***	0.47 (0.24)	0.94 (0.02) **	0.88 (0.04)	0.41 (0.16)
	BiLSTM	0.91 (0.03) ***	0.94 (0.02) ***	0.4 (0.14)	0.94 (0.02) ***	0.89 (0.04)	0.3 (0.23)
	CNN	0.94 (0.02) ***	0.97 (0.01) ***	0.49 (0.21)	0.94 (0.03) ***	0.93 (0.03) ***	0.43 (0.34)
	TCN	0.97 (0.01) ***	0.95 (0.02) ***	0.56 (0.09)	0.95 (0.01) ***	0.96 (0.02) ***	0.51 (0.2) *
	ResNet	0.91 (0.04) ***	0.96 (0.01) ***	0.41 (0.41)	0.97 (0.01) ***	0.93 (0.01) ***	0.55 (0.16)
Ensemble	RF	0.83 (0.06)	0.86 (0.04)	−0.01 (0.24)	0.86 (0.03)	0.86 (0.04)	0.14 (0.5)
	GBM	0.92 (0.03) ***	0.93 (0.02) ***	0.63 (0.1)	0.96 (0.01)	0.93 (0.01) ***	0.54 (0.18)

*** p<0.001, ** p<0.01, * p<0.05. Data are expressed as median (median absolute deviation).

## Data Availability

The raw data supporting the conclusions of this article will be made available by the authors on request.

## Abbreviations

The following abbreviations are used in this manuscript:

CVD	Cardiovascular disease
PPO	Peak power output
IMU	Inertial measurement unit
BiLSTM	Bidirectional long short-term memory
CNN	Convolutional neural network
ReLU	Rectified linear unit
ResNet	Residual neural network
TCN	Temporal convolutional network
RF	Random forest regressor
GBM	Gradient boosting machine
MdAPE	Median absolute percentage error
